# The network structure affects the fixation probability when it couples to the birth-death dynamics in finite population

**DOI:** 10.1371/journal.pcbi.1009537

**Published:** 2021-10-27

**Authors:** Mohammad Ali Dehghani, Amir Hossein Darooneh, Mohammad Kohandel

**Affiliations:** 1 Department of Physics, University of Zanjan, Zanjan, Iran; 2 Department of Applied Mathematics, University of Waterloo, Waterloo, Canada; University of Sheffield, UNITED KINGDOM

## Abstract

The study of evolutionary dynamics on graphs is an interesting topic for researchers in various fields of science and mathematics. In systems with finite population, different model dynamics are distinguished by their effects on two important quantities: fixation probability and fixation time. The isothermal theorem declares that the fixation probability is the same for a wide range of graphs and it only depends on the population size. This has also been proved for more complex graphs that are called complex networks. In this work, we propose a model that couples the population dynamics to the network structure and show that in this case, the isothermal theorem is being violated. In our model the death rate of a mutant depends on its number of neighbors, and neutral drift holds only in the average. We investigate the fixation probability behavior in terms of the complexity parameter, such as the scale-free exponent for the scale-free network and the rewiring probability for the small-world network.

## Author summary

In this work, we examine an evolutionary model that considers the effect of competition between the mutated individuals for acquiring more resources. This competition has an effect on the death rate of mutants. The model purposes that the death rate of each mutant depends on the number of its neighbors, while the average death rate in the population is equal to one. The birth rate for all individuals is assumed to be the same and equal to one. This situation is called here the ‘neutral drift in average’. We study the dynamics of the model on complex networks to take into account the non-uniformity of the environment. The results show the fixation probability differs from the Moran model. For the construction of this model, we were biologically motivated by the avascular tumour, which consists of a population of normal and cancer cells. The cancer cells likely need more oxygen than normal cells. There is a competition between cells for consuming oxygen, and cancer cells are far more sensitive to the amount of oxygen in the environment than normal cells. This means the death rate of a cancer cell grows by increasing the number of its neighbors.

## Introduction

Evolutionary dynamics is the study of the mathematical principles governing the evolution of biological organisms. It studies the variation of the population size according to the reproduction, mutation and selection processes. In an asexual population a member reproduces a copy of itself when it becomes mature without needing any other members. The member can also be mutated if this process involves mistakes, or under the influence of environmental factors. The mutated member has a chance to fixate in the population if it has acquired the qualities to make it more capable of reproduction and/or resistant to death. In evolutionary dynamics, we define a quantity called fitness to include all these qualities. Therefore, the fitness function may depends on the birth rate and/or the death rate of individuals.

Population structure affects the outcome of ecological and evolutionary dynamics. Evolutionary graph theory provides a useful model for representing population structure [1]. In this approach, the population members are considered as the graph’s nodes, and the edges of graph represents the interaction between them [1]. At each step of evolution, a member may die, reproduce or undergo no changes. The neighbours of a member on a graph determine the probability for its dying or reproducing. In a constant size population, the important question is how the phenotypic mutation in a member can be fixed during the evolutionary process. The fixation process is characterized by knowing two quantities, the probability and the time required for fixation when the process starts with one mutant in the population. Over the past two decades, many studies have investigated the effect of graph structures on the fixation probability and/or fixation time [2–12]. In addition, it has been shown that other factors such as the initial location of the mutant on a graph can affect the fixation probability [1, 13–17].

A significant result in the graph based evolutionary dynamics is the isothermal theorem. It states that the fixation probability of mutants in a large group of graph structures (known as isothermal graphs, which include regular graphs) coincides with that for the mixed population. The Moran model is one of the well-known and essential process used in the study of evolutionary dynamics [18]. In this model, we deal with a constant population of *N* members and two types of individuals (or cells), the wild type and the mutant. The sizes of sub-populations are changed by passing through the time. At each time step, there are two possible scenarios for the Moran process. In a birth–death (BD) process (or the invasion process), a birth event is followed by a death event, and in a death–birth (DB) process (or voter models), a death event happens first. Kaveh et al. [19] examined the extension of the isothermal theorem for the BD and DB models. They proved that for general BD and DB processes with arbitrary birth and death rates of mutants, the fixation probabilities of mutants can be different from those obtained in the mass-action populations. In the case that “self” is included in the neighborhood graph, the BD and DB processes are identical, and the probability of fixation is given by:

$$\begin{array}{c} \rho_{1}=\frac{1-d/r}{1-{\left(d/r\right)}^{N}} \end{array}$$ (1)

where *d* = *d*_*B*_/*d*_*A*_ and *r* = *r*_*B*_/*r*_*A*_. Here, *d*_*B*_ (*r*_*B*_) and *d*_*A*_ (*r*_*B*_) are death (proliferation) rates for mutant (B) and wild type (A). However, if “self” is not included, then the BD and DB processes are not the same, and the difference is of the order of 1/*N*. If *r* = 1 (or *d* = 1) for the DB (or BD) process, Eq (1) holds, which can be interpreted as the isothermal theorem for the mass action scenario. The isothermal theorem was initially proven in [1] to hold for the *d* = 1 BD update, and Kaveh et al. [19] showed that it also holds for the *r* = 1 DB update.

Moreover, the fitness of a species may vary in space and time due to the influence of environment. In particular, mutant evolution in random environments has been studied by evolutionary biologists and and mathematicians for decades. It has been shown that both fixation probability and fixation time are affected due to randomly fluctuating environments, even for the neutral drift [20–22]. The fixation probability and fixation time depend on the variability of fitness or its statistics [23–29]. Recent studies on social networks and biological systems have investigated these kind of models as well [30–32].

In this paper, we intend to study the evolutionary process on complex networks and couple its dynamics to the topology (or the structure) of the networks. We consider a network containing mutants and wild types. Each node in the network is connected to a different number of other nodes. The fitness of both mutants and wild types are one, and the relative death rate, *d*, is considered to be equal to its normalized degree, that is, the degree divided by the average degree (i.e. the average number of neighbors for any node). The reason for this choice is we want to consider the case of the neutral drift condition, in which the average relative death is one (the death rate for the wild type is one). This is a kind of generalization of the neutral drift which have been previously presented in literature [20, 22, 29]. At the first step, we examine this model analytically for a star graph that has a simple structure, and then solve it numerically. Then, we use simulations to study this model for networks with more complex structure such as small-world and scale-free networks. Complex networks are realistic graph structures observed in the nature that simultaneously included both the randomness and the regularity in the structure [33, 34].

## Model

Complex networks and the average neutral drift are two topics that distinguish this work from other works. In this section we explain these topics, the first one in brief because of the existence of a large amount of literature on this topic, but the latter one needs more explanation. After elaborating on these concepts, we describe the simulation algorithm which we use to implement the model. At the end of this section, some elucidating points about this model are discussed.

### Complex networks

Nowadays, the complex networks theory appears as a useful method for studying many phenomena in various disciplines of science and technology [35–38]. There are many systems in nature which can be modeled by complex networks such as neural networks [39], cells [40], internet [41, 42], earthquakes [43–48] and climate network [49–51]. Every network is characterized by knowing the nodes and how they are connected to one other. For example, the brain is a network which its nodes are neurons and synapses connect the neurons together [52].

In many natural and technical systems, it is very difficult to quantitatively understand the interaction between different system’s parts. The complex networks are a simple way to model these systems without knowing details of interactions. The network properties, like the degree distribution function, clustering coefficient, and average shortest path length can describe the system on the whole [53]. The degree distribution function, *p*(*k*) is the most important quantity that is used to classify network topologies (or structures). It shows the probability of finding a node in the network with *k* neighbors. In practice, we use the following method to compute the degree distribution function:

$$\begin{array}{c} p\left(k\right)=N_{k}/N \end{array}$$ (2)

where *N*_*k*_ is the number of nodes with *k* neighbors and *N* is the total number of nodes. According to the degree distribution, we can distinguish two categories of networks. First, networks with power law degree distribution, *p*(*k*) ∼ *k*^−*γ*^, are called scale-free networks. In such networks, it is very likely to find a node with a large number of neighbors. A node with high degree is called a hub. The power law exponent *γ* is mathematically more than one but for most of the real networks is bounded between 2 and 3. The existence of growth and preferential attachment mechanism are two major reasons for the emergence of scale-free networks. The internet is an instance for these kind of networks [54].

The second category of networks have Poisson (exponential) degree distribution, *p*(*k*) ∼ exp(−*k*/*k*_0_). Nodes with approximately *k*_0_ neighbors are the most frequent nodes in this type of networks. Hence, we call these networks homogeneous, unlike the scale-free networks that are heterogeneous.

The clustering coefficient measures the tendency of nodes to make a cluster together in a network. In a graph, a triangle is created when two neighbors of a node are neighbors with each other. This situation may not happen for all neighbors. Therefore, among all possible triangles, only some of them may actually exist in the network. For a node, the clustering coefficient is defined as the number of existing triangles to all possible triangles which this node is common for them. The clustering coefficient of the network is derived by averaging over all node’s coefficients. In a random network, which is constructed by connecting nodes randomly, the clustering coefficient is very small in comparison with regular networks with the same number of links and nodes. In a regular network, all nodes have the same degree.

The minimum number of edges for traversing between two nodes is called geodesic. Averaging this quantity over all pairs in network gives the average shortest path length, which is a characteristic for the network size. Random networks have small value of the average shortest path length, but it is opposite for the regular networks.

In a wide variety of the real networks like the electrical power grid, we observe that they have small value of the average shortest path length, but a large clustering coefficient in comparison with the random networks. This category of networks have the small world property, or they are simply called small-world networks [55]. A small world network can be constructed by random rewiring edges of the regular networks. These kind of networks have Poisson (exponential) degree distribution in the large number of nodes limit.

In S1 Appendix the properties of main network classes are shown in brief. This classification is based on three characteristics of networks. It is possible to categorize networks in terms of other properties. In addition there are several hybrid models which incorporate some of these characteristics together.

### Neutral drift in average

Consider a constant population with *N* members, which consists of two types of species, A (wild type) and B (mutant). This population evolves under a BD Moran process, in which the fitness depends on the death rate. There are two fixed points for this process, i.e. the states of system that all members in the population become identical and then there is no change in the system. When the process starts with one mutated member in the population, it could finally be in the state with all members are mutants, i.e., mutation is fixed. The probability for this event happening is called fixation probability. In the case of neutral drift the wild type and mutant species have similar fitness and there is no preference between them. The fixation probability for mutation under neutral drift condition is equal to inverse of the population size, 1/*N*.

The environment and time may have an effect on the fitness of the species. In this case, we need to extend the meaning of similarity for the species’ fitness. Thus, we assume that some mutants have larger fitness than the wild types and others vice versa, while on average, they are the same. This situation is called the average neutral drift, and it is interesting to find a relation between the fitness variance and fixation probability [20].

In reality, fitness is influenced by the birth and death rates, which may vary for various members due to their access to vital resources. For example, if a cell has a large number of neighbour cells, then its death rate increases since there will be a competition for space and nutrients. In this work, we assume the fitness variance arises from a different number of neighbours for each member or, in other words, the population structure. In such conditions, we can use the concept of average neutral drift and explore the effect of variance in the number of neighbours on the fixation probability.

In the following we focus on the BD process on heterogeneous networks. This process consists of two main steps: in the beginning, an individual is randomly chosen among the population for reproduction, then one of its neighbors are selected at random for dying according to their death rate. We consider the same value for the birth rate of all members, regardless of whether it is a wild type (A) or mutant (B), *r*_*A*_ = *r*_*B*_ = 1. The death rate for all wild types are equal to one independent of their location on the network, *d*_*A*_ = 1. However, for mutants, the death rate depends on the number of its neighbors. If the mutant is located on the *i*-th node, its death rate is, $d_{B}^{\left(i\right)}=k_{i}/<k>$, where *k*_*i*_ is used for the degree of *i*-th node and < *k*> is the average degree of the network. It is clear that the average death rate of mutants is equal to one, < *d*_*B*_ > = 1. By choosing such a distribution for the death rates, we couple the dynamics of the system to the structure of the network. This is a novel way to apply heterogeneity in the system if we consider the evolution process on the heterogeneous networks.

**Fig 1:**
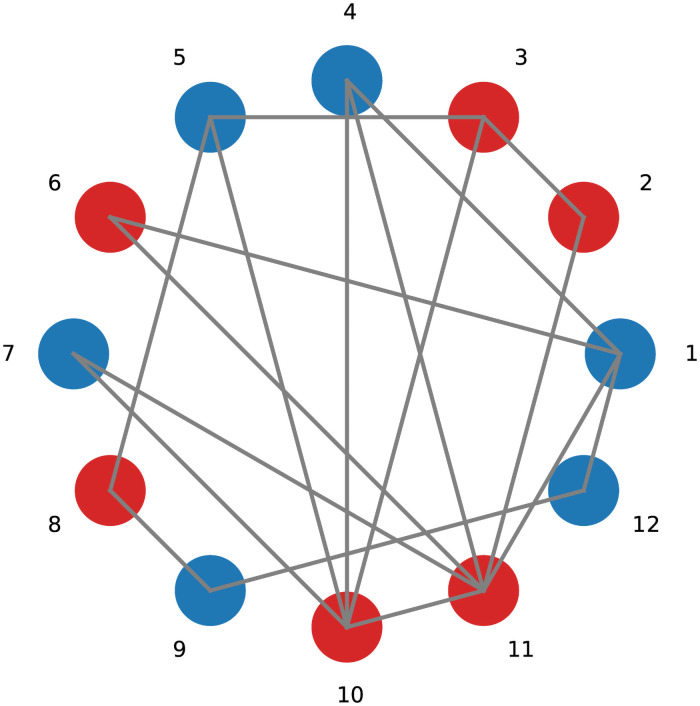
General graph. A graph with 12 nodes which some of them are occupied by wild-types (blue circles) and the others by mutants(red circles).

To understand the above model more clearly, we have sketched an example of such a network in Fig 1. The node 11 could be selected randomly with a probability 1/12 for reproduction. Its offspring will be placed in one of the neighboring nodes, so one of the neighbors should be selected to die according to its death rate. The node 11 has six neighbors, {1, 2, 4, 6, 7, 10}. Three nodes among them are wild type, {1, 4, 7}, and have the death rate equal to one. The nodes {2, 6, 10} are mutants with degree *k*_2_ = 2, *k*_6_ = 2 and *k*_10_ = 5. The average degree for this network is, < *k* > = 3, then the death rates for mutants are: $d_{B}^{\left(2\right)}=2/3,d_{B}^{\left(6\right)}=2/3$ and $d_{B}^{\left(10\right)}=5/3$. Now, the probability of placing the offspring in these neighboring nodes are *p*_1_ = 1/6, *p*_2_ = 1/9, *p*_4_ = 1/6, *p*_6_ = 1/9, *p*_7_ = 1/6 and *p*_10_ = 5/18.

### Simulation algorithm

**Algorithm 1: Simulation Steps** Simulation steps for the birth-death (BD) process

**Data**: Number of Nodes, Average Degree, Rewiring Probability or Scale-Free Exponent and Ensemble Size

**Results**: Fixation Probability

Number of Succession = 0;

**for** *s* ← 1 **to** *Ensemble Size* **do**

 -Generate network with predefined number of nodes, average Degree and rewiring probability or scale-free exponent;

 -Set birth rate equal to one for all nodes;

 -Determine death rate for all nodes (if node has wild type state it is equal to one and for mutant state it depends on the number of neighbors);

 -Set the state of all nodes as wild type;

 -Choose randomly a node and set its state as mutant;

 **while** *Number of mutant is not equal to zero or equal to the number of nodes*

  **do**

  -Choose randomly a node for reproduction;

  -Choose randomly one of its neighbors for dying according to their death rate;

  -Change the state of dead node to the state of reproducing node;

 **if** *Number of mutants is equal to the number of nodes* **then**

  -Increase number of succession by one;

-Fixation probability is equal to number of succession dividing by the ensemble size;

In most cases, where we have an unstructured network with a large number of nodes, the simulation is the best and sometimes the only way to compute the fixation probability. The simulation has a very straightforward algorithm. However, it may be time-consuming in the absence of any symmetry in the network.

At the first step, we must generate a network with predefined characteristics such as the number of nodes, the average degree and the rewiring probability (scale-free exponent). Then, we assign to each node four quantities: the wild-type birth rate, the wild-type death rate, the mutant birth rate and the mutant death rate. These quantities remain unchanged until the end of the simulations. In our model, these quantities for the *i*-th node are {1, 1, 1, *k*_*i*_/< *k* >}, where *k*_*i*_ stands for the node degree and < *k* > describes the average degree of the network. We also attribute a state variable to each node, which shows it is occupied by a wild-type or a mutant. Initially, all nodes are wild-type except one that is randomly selected as a mutant. The simulation begins by randomly choosing a node for reproduction. Then, one of its neighbors is randomly selected for dying according to their death rate. The state of the dead node changes to the state of the mother node. This process is repeated until the state of all nodes is the same. We should repeatedly run this process and count the number of times which all nodes have the mutant state, i.e. the number of successions. The fixation probability is obtained by dividing this number by the number of runs. A simple pseudocode for this simulation is written in Algorithm 1.

### Relation to biological systems

Recently, many authors have been interested in studying the evolutionary dynamics in the heterogeneous population [3, 20, 22, 29]. Here, we look at this issue from a new perspective.

In realistic cases, the resources are distributed irregularly in an environment. Those species that have access to a resource are assumed to be neighbors with each other. The distinct resources may also have different volumes, which implies heterogeneity in the environment. The non-uniformity in the pattern of resources in an environment results in the complex structure in the neighborship of species which is represented by a complex network. The small-world network is a suitable candidate for studying the effect of randomness in the population structure; by increasing the rewiring parameter, we can construct networks with a high amount of randomness from a regular graph to a totally random graph. The heterogeneity in the population is modeled by using the scale-free networks. The scale-free exponent can be considered as a measure for the network heterogeneity, and we reach a random graph by increasing this exponent.

Limitation in the volume of resources causes the species to compete with one another. Therefore, the number of neighbors becomes an important factor in the population dynamics in a system. In other words, the evolution process is coupled to the network structure.

The neutrality assumption guarantees that there is no bias in the species’ fitness for domination in the population. We try to keep this assumption even statistically; for this purpose, the average neutral drift is defined.

One example of this model is the competition between cancer and normal cells in an avascular tumour. Most solid tumours first go through an avascular state, up to a maximum size of about 1–2 mm in diameter, before the lack of oxygen and other essential nutrients prevent further growth. In avascular tumours, cancer cells (mutants) need more oxygen than normal cells (wild-types). There is a competition between cells for consuming oxygen, and cancer cells are more sensitive to the amount of oxygen in the environment than normal cells. If the number of neighbors is large, each cell’s share decreases, and then the death probability (rate) for cancer cells grows [56, 57]. The selection of network class for a tumour depends on the chemo-physical properties of the extra-cellular matrix and how the cells are distributed on it. In addition, what the resource is that they compete for it. For example, if the cells compete for a nutrient that spreads in a limited range, then a few numbers of cells can be connected to each other in the vicinity of a resource. Therefore, we do not have any node with a high number of connections, and a small-world network is better for modeling than a scale-free network.

## Results and discussions

Writing a master equation for an arbitrary grid is not easy in most cases and we need to use numerical simulations for computing quantities such as the fixation probability. In the next part, we first write a master equation for the simple heterogeneous network of the star graph [11]. We solve the equations numerically and compare the solutions with the simulation results to ensure about the simulation accuracy.

### Average neutral drift for star graph

In this section, we examine the BD process for star graphs, assuming the death rate of each mutant depends on the degree of the node in which it is located, as we described in the previous section.

A star graph is a graph with a central core and *n* leaves. The degree of the central core is, *k*_*o*_ = *n* and all leaves have degree equal to one, *k* = 1. The average degree of graph is < *k* > = 2*n*/(*n* + 1). Then, by simple calculation we can find the death rate for a mutant in the core node, *d*_*o*_ = (*n* + 1)/2, and for leaves *d* = (*n* + 1)/2*n*.

**Fig 2:**
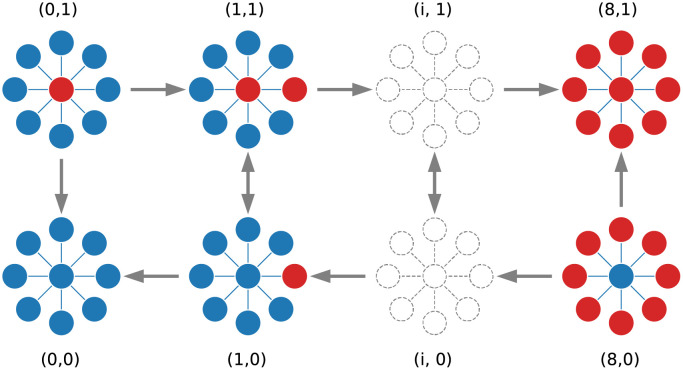
Configurations of star graph. A star graph with *N* = 8 + 1 nodes. The diagram shows all possible configurations for the star graph. An ordered pair, (*u*, *v*), indicates a configuration exclusively wherein *u* is the number of mutants located on the leaves, and *v* shows whether the core node is occupied by a mutant or not. The wild-type and mutant are distinguished by blue and red colors respectively. The arrows exhibit the allowed transitions between these configurations.

There are 2(*n* + 1) configurations according to the position of mutants on the graph. We denote each configuration by an ordered pair, (*u*, *v*), wherein the first element in pair is the number of mutants located on leaves. This number varies between 0 and *n*. The second element indicates whether the core node is occupied or not. It can be 0 or 1. It is obvious that the number of mutants comes from the sum of these numbers. In the BD process, only transitions between some of these configurations are allowed; those transitions that lead to an increase or decrease in the number of mutants by one. In Fig 2, we show the possible configurations and the allowed transitions between them for a star graph with 8 leaves.

By simple algebraic manipulations, we can calculate the probability for allowed transitions in the BD process. They could be classified as follows:

$$\begin{array}{c} \begin{array}{cc} & W\left(\left(i,0\right)\to \left(i-1,0\right)\right)\equiv \alpha_{i}^{0}=\frac{1}{n+1}\cdot \frac{id}{id+(n-i)}\\ & W\left(\left(i,0\right)\to \left(i,1\right)\right)\equiv \beta_{i}^{0}=\frac{i}{n+1}\\ & W\left(\left(i,0\right)\to \left(i,0\right)\right)=1-\alpha_{i}^{0}-\beta_{i}^{0}\\ & W\left(\left(i,1\right)\to \left(i+1,1\right)\right)\equiv \alpha_{i}^{1}=\frac{1}{n+1}\cdot \frac{n-i}{id+(n-i)}\\ & W\left(\left(i,1\right)\to \left(i,0\right)\right)\equiv \beta_{i}^{1}=\frac{n-i}{n+1}\\ & W\left(\left(i,1\right)\to \left(i,1\right)\right)=1-\alpha_{i}^{1}-\beta_{i}^{1} \end{array} \end{array}$$ (3)

Two coupled equations which explain this process are,

$$\begin{array}{c} \begin{array}{cc} & \pi_{i}^{1}=\alpha_{i}^{1}\pi_{i+1}^{1}+\beta_{i}^{1}\pi_{i}^{0}+\left(1-\alpha_{i}^{1}-\beta_{i}^{1}\right)\pi_{i}^{1}\\ & \pi_{i}^{0}=\alpha_{i}^{0}\pi_{i-1}^{0}+\beta_{i}^{0}\pi_{i}^{1}+\left(1-\alpha_{i}^{0}-\beta_{i}^{0}\right)\pi_{i}^{0} \end{array} \end{array}$$ (4)

Here, $\pi_{i}^{1}$ is the fixation probability when we begin with *i* mutants located on the leaves, and a mutant is placed in the central core as well. $\pi_{i}^{0}$ is the fixation probability by starting from the *i* mutants are on leaves, and in the central core a resident exists.

By introducing the following parameters,

$$\begin{array}{c} \begin{array}{cc} & P_{i\leftarrow }=\frac{\alpha_{i}^{0}}{\alpha_{i}^{0}+\beta_{i}^{0}}=\frac{id}{id+i(id+n-i)}\\ & P_{i↓}=\frac{\beta_{i}^{0}}{\alpha_{i}^{0}+\beta_{i}^{0}}=\frac{id+n-i}{d+id+n-i}\\ & P_{i\to }=\frac{\alpha_{i}^{1}}{\alpha_{i}^{1}+\beta_{i}^{1}}=\frac{1}{1+id+n-i}\\ & P_{i↑}=\frac{\beta_{i}^{1}}{\alpha_{i}^{1}+\beta_{i}^{1}}=\frac{id+n-i}{1+id+n-i} \end{array} \end{array}$$ (5)

into Eq (4), and performing simple algebraic manipulations, the master equation can be written in the matrix form *X*_*i*_ = *A*_*i*_ *X*_*i*−1_:

$$\begin{array}{c} \left[\begin{array}{c} \pi_{i}^{1}\\ \pi_{i}^{0} \end{array}]=[\begin{array}{cc} \frac{1}{P_{i-1\to }} & -\frac{P_{i-1↑}}{P_{i-1\to }}\\ \frac{P_{i↓}}{P_{i-1\to }} & \left(P_{i\leftarrow }-\frac{P_{i-1↑}P_{i↓}}{P_{i-1\to }}\right) \end{array}][\begin{array}{c} \pi_{i-1}^{1}\\ \pi_{i-1}^{0} \end{array}\right] \end{array}$$ (6)

It is clear that *X*_*n*_ = *A*_*n*_ *A*_*n*−1_ ⋯ *A*_1_ *X*_0_. Then, by applying the boundary conditions, $\pi_{0}^{0}=0$ and $\pi_{n}^{1}=1$, we arrive at the following equation:

$$\begin{array}{c} \left[\begin{array}{c} 1\\ \pi_{n}^{0} \end{array}]=(\prod_{i=1}^{n}\;[\begin{array}{cc} \frac{1}{P_{i-1\to }} & -\frac{P_{i-1↑}}{P_{i-1\to }}\\ \frac{P_{i↓}}{P_{i-1\to }} & \left(P_{i\leftarrow }-\frac{P_{i-1↑}P_{i↓}}{P_{i-1\to }}\right) \end{array}])[\begin{array}{c} \pi_{0}^{1}\\ 0 \end{array}\right] \end{array}$$ (7)

**Fig 3:**
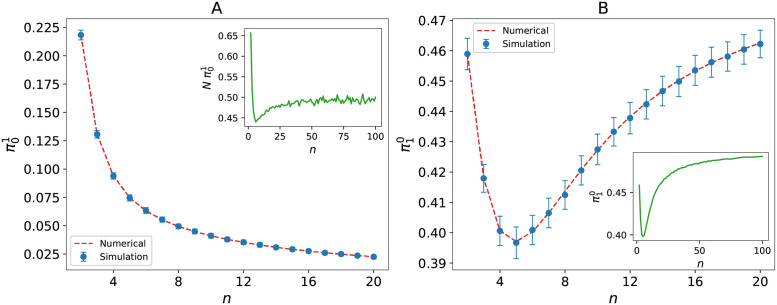
Fixation probability of star graph. Fixation probability of the star graph, obtained numerically and by simulation based on our model: (A) The mutant is located on the core node, the inset shows the ratio of the fixation probability for this configuration to the Moran model. (B) The mutant is located on one of the leaves, the inset shows its saturation for large number of leaves. The simulation runs 10^6^ times for each point, and the error bars represent the standard deviation of simulation results.

This equation can be numerically solved to find $\pi_{0}^{1}$ and $\pi_{n}^{0}$. Then, by using Eq (6), the other $\pi_{i}^{1}$ and $\pi_{i}^{0}$ are successively obtained. In Fig 3, the fixation probabilities $\pi_{0}^{1}$ and $\pi_{1}^{0}$ are plotted in terms of *n*, the number of the leaves. The simulation result is in good agreement with the numerical solution. We explain the simulation steps in detail below.

As observed in Fig 3, the $\pi_{0}^{1}$ decreases more rapidly than the Moran model at first, but the distance between two curves is reduced by increasing the number of leaves very slowly and finally reaches a constant distance. This is an expected behavior because the leaves are not connected to each other and if a mutant is located on a leaf it cannot put its offspring on the other leaves directly. It should first occupy the core node and then reach to the other leaves. This two-step process depends on the death rate of mutant in the core node which is increased with *n*. This means the process of spreading mutant between leaves is facilitated by increasing the number of leaves, and therefore the difference with the Moran model becomes constant.

By using Eqs (5) and (6), it can be proved analytically that $\pi_{1}^{0}=\left[\left(n+1\right)\left(2n^{2}+1\right)/\left(2n^{2}+2\right)\right]\;\pi_{0}^{1}$. The behavior of $\pi_{1}^{0}$ is determined by the competition between decreasing function $\pi_{0}^{1}$ and the increasing coefficient that approaches *n* + 1 for large *n*. As it is shown in Fig 3, $\pi_{1}^{0}$ has a minimum at *n* = 5 and then saturates for large *n*.

### Simulation results for complex networks

For larger and more complicated graphs the simulation is an affordable or only way for computing the fixation probability. In this section, we focus on simulation of the BD process on complex networks.

There are two important classes of networks, the small-world and the scale-free networks. The small-world networks are placed between the regular and random graphs. For constructing a small-world network, we begin with a regular graph with the same number of nodes and links. Then, all the links are rewired by the probability *q*. For *q* = 0, the network remains regular and with *q* = 1 we generate a random graph. The middle values of *q* result in the small-world networks. The properties of such networks depend on the rewiring probability *q*. The scale-free network is constructed dynamically. We begin with one node, or a small-size regular graph, and then add new nodes successively. The new node preferentially links to the previous nodes with higher degree. Thus, some nodes become a hub with a degree more than the average. The degree distribution of such networks has a power-law relationship and the value of the exponent characterizes them. In the following, we study the effect of rewiring probability and the scale-free exponent on the fixation probability.

**Fig 4:**
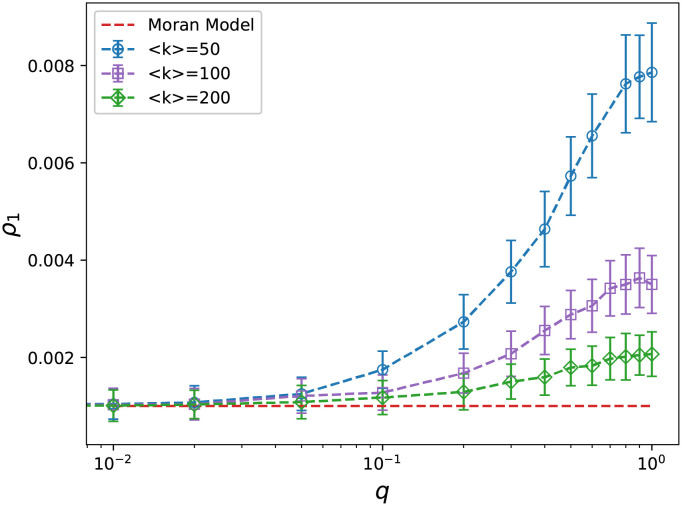
Fixation probability of small-world networks. Fixation probability of the small-world networks with 1000 nodes in terms of the rewiring probability. The Moran model (dashed line) is plotted for the comparison. Fixation probability increases smoothly by increasing the rewiring probability. The increase is proportional with inverse of the average degree. (The error bars represent the standard deviation of simulation results).

First, we examine the effect of rewiring probability *q* on the fixation probability *ρ*_1_. For this purpose, we consider different values for *q* in the interval [0, 1]. Then, for each value of *q*, the simulation is run 10000 times for 100 different networks with similar structure; the same number of nodes and the average degree, then the fixation probability is computed as the final result. Fig 4 shows the obtained results. When *q* = 0, we have an exact regular graph. In this case, the degree of all nodes are the same and equal to the average degree, so the death rate is equal to one for all of them. For a regular graph with *N* nodes, the fixation probability is 1/*N* and identical to the fixation probability for the Moran model. By increasing *q*, the fixation probability is also increased. The reason is that rewiring creates shortcuts between nodes. In addition, *ρ*_1_ decreases by increasing the average degree. To justify these results, it is worth nothing that the degree distribution for small world networks is similar to Poisson which is skewed for small degrees, and therefore in the selection of neighbor species for death, the mutants are less likely to be in average chosen because their death rates are less than one. Furthermore, by increasing the average degree, the Poisson distribution approaches a Gaussian distribution which is symmetric, and thus it is more likely to choose a mutant for death, resulting in a decrease in the fixation probability. In the extreme case of very large < *k* >, we have a complete graph and so the fixation probability is obtained by the the Moran model.

It should be noted that for non-uniform systems, the fixation probability depends on the standard deviation of fitness [20, 22]. Here, in our model, the fitness of mutants is only a function of their death rate, and the death rate itself depends on the node’s degree that a mutant occupies. This means the fixation probability is function of the degree fluctuation, i.e. $\Delta =\sqrt{\frac{<k^{2}>}{<k{>}^{2}}-1}$. In mathematical view, the degree distribution of the small-world network is a combination of several Poisson distributions with coefficients that are functions of the rewiring probability [58]. Although an exact form for the average degree or the fluctuation does not exist, we can realize numerically that the fluctuation is ascending by increasing the rewiring probability and descending by growing the average degree. This is the same behavior that we observe in the results.

The behavior of the fixation probability for the small world shows that randomness in the environment and the sparse neighborship structure are favored for the mutants to be dominant in a population. Here *q* measures the randomness, and the sparsity of the structure is demonstrated by the average degree.

**Fig 5:**
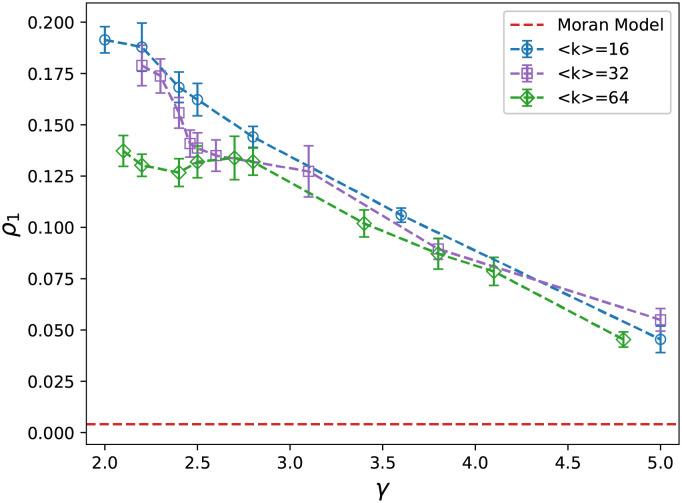
Fixation probability of scale free networks. Fixation probability of the scale-free networks with 256 nodes in terms of the scale free exponent. The fixation probability decreases by increasing the scale-free exponent, however, different behaviors are observe in two regions; 2 < *γ* ≤ 3 and *γ* > 3, regarding to the average degree. (The error bars represent the standard deviation of simulation results).

The result for the fixation probability on scale-free networks are plotted in Fig 5. We construct 100 networks with the same number of nodes and the average degree then repeat the simulation 2560 times for each distinct network. The two regions are distinct: 2 < *γ* ≤ 3 and *γ* > 3. The latter shows networks with a finite value of the degree standard deviation [59]. The existence of a node with degree less than the average is more probable because of the decreasing behavior of the power law function. The mutant benefits from this fact, and thus we expected the fixation probability to be greater than the Moran model. When the scale-free exponent is increased the standard deviation becomes narrower and therefore the probability of selection of mutant for death is close to the wild type. This result in a decrease in the fixation probability toward the value of Moran model by increasing the scale-free exponent. When we fix the minimum degree of the network, the standard deviation of the degrees only depends on the scale-free exponent, hence the fixation probability does not depend on the average degree for *γ* > 3.

For 2 < *γ* ≤ 3, by the same reason as mentioned above, the mutants benefit from the totally skewed distribution of nodes degree to fixate in the system. However, in this case, the standard deviation of degrees depends on both the scale-free exponent and the size of the network. As a consequence, we observe that the fixation probability depends on the average degree as well as the scale-free exponent.

The existence of hubs, nodes with a high degree, is the reason for the heterogeneity of a network. The probability of finding a hub decreases when the scale-free exponent becomes greater. Therefore, we can consider the inverse of *γ* as an indicator for heterogeneity. The results show that mutants benefit from the system’s heterogeneity and randomness, which we have discussed previously.

As we mentioned above, the fixation probability in heterogeneous systems depends on the standard deviation of death rates [19, 20, 22]. It is obvious that this argument leads to dependency of the fixation probability on the fluctuation of the nodes degree. For the region *γ* > 3, the degree fluctuation is equal to $\sqrt{\frac{{\left(\gamma -2\right)}^{2}}{\left(\gamma -1)(\gamma -3\right)}-1}$ [59] which is only function of *γ*. While in the region 2 < *γ* < 3 it is $\Delta =\sqrt{\frac{{\left(\gamma -2\right)}^{2}}{\left(\gamma -1)(\gamma -3\right)}\left(1-{\left(\frac{\gamma -1}{\gamma -2}\frac{k_{max}}{<k>}\right)}^{3-\gamma }\right)-1}$ where *k*_*max*_ is the largest degree in the network which scales with the network size (see S2 Appendix).

## Conclusion

In this work, we defined the concept of neighborship between species in terms of accessing a resource. This kind of definition differs from the common view that takes into account the proximity of a neighbor. The randomness and heterogeneity in the distribution of resources in an environment result in a complex pattern of relation between species. The neighborship pattern can be represented mathematically by a complex network. Here, we proposed a model for the BD process on complex networks to study the influence of the environmental irregularity on the fixation of mutant species. We assumed that the death rate for a mutant depends on the number of neighbors while the other parameters were equal to one. The most significant contribution of the current work was the coupling between dynamics and structure, resulting in a violation in the isothermal theorem. We examined the model numerically for the star graph and used the simulation for the two main categories of complex networks, i.e. small-world and scale-free networks. We obtained that the fixation probability for the star graph is less than the complete graph (the Moran model), but their different approaches to a constant value for a large number of leaves. The results for the small-world network exhibited that by increasing the rewiring probability, the fixation probability increases in comparison to the complete graph. This increase becomes negligible for the large values of the average degree. It was found that the fixation probability for the scale-free decreases and approaches the value for the complete graph by increasing the scale-free exponent. Two regions could be distinguished: *γ* > 3, where all networks with the same number of nodes behave similarly despite the average degree, while in the interval 2 < *γ* < = 3 the fixation probability depends on the average degree as well as the scale-free exponent. All these results can be justified by knowing that the fixation probability depends on the fitness standard deviation, which is tied to the degree fluctuation in the network.

Generally, it can be deduced that the mutants benefit from the environment’s non-uniformity. The randomness and heterogeneity help the mutant to fixate in a system with higher probability.

It would be interesting to investigate the fixation time in this model, and also to study the other implication of the model such as finding the survivorship curve for some categories of organisms.

## Supporting information

S1 Appendix Main network classes. (PDF)

S2 Appendix Mathematical notes on the scale free networks. (PDF)
