# Supplementary material for: The network structure affects the fixation probability when it couples to the birth-death dynamics in finite population
Source: PLoS Comput Biol. 2021 Oct 27;17(10):e1009537. doi: 10.1371/journal.pcbi.1009537 (PMC8575310; doi:10.1371/journal.pcbi.1009537)
Supplement: S1 Appendix — (PDF) [file pcbi.1009537.s001.pdf]

S1 Appendix. Main Network Classes

	Regular	Small World	Random	Scale Free
Network Plot	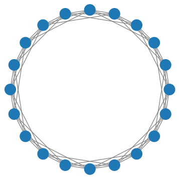 $q = 0$	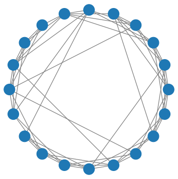 $0 < q < 1$	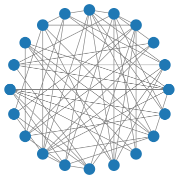 $q = 1$	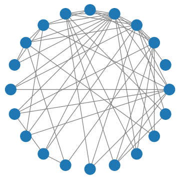 $\gamma > 2$
Degree Distribution	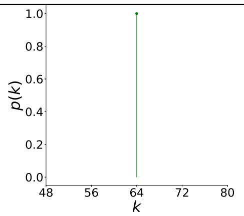 Kroneker delta	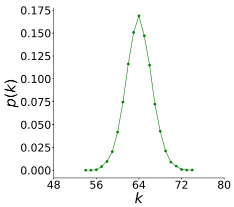 Poisson Like	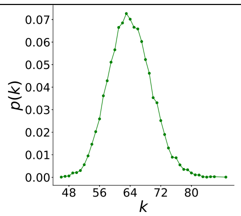 Poisson	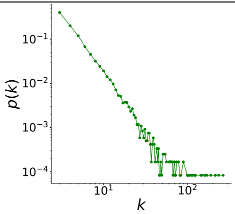 Power law
Clustering Coefficient	$\frac{3(<k>-2)}{4(<k>-1)}$	$\frac{3(<k>-2)}{4(<k>-1)}(1-q)^{3}$ *	$\sim \frac{<k>}{N}$	$N^{-\frac{3}{4}}$ **
Average Shortest Path Length	$\sim \frac{N}{<k>}$	$\sim \frac{\ln(N<k>q)}{<k>^{2}q}$ *	$\sim \frac{\ln N}{\ln <k>}$	$\frac{\ln N}{\ln \ln N}$ **

Each network plot consists of 20 nodes and 60 links, while for plotting the degree distribution we use network with 10000 nodes. q demonstrates the rewiring probability and γ is the scale free exponent. We use N and $< k >$ for the number of nodes and the average degree respectively.

* For Watts-Strogatz model.

** For Barabasi-Albert model.
