# Supplementary material for: The network structure affects the fixation probability when it couples to the birth-death dynamics in finite population
Source: PLoS Comput Biol. 2021 Oct 27;17(10):e1009537. doi: 10.1371/journal.pcbi.1009537 (PMC8575310; doi:10.1371/journal.pcbi.1009537)
Supplement: S2 Appendix — (PDF) [file pcbi.1009537.s002.pdf]

S2 Appendix. Mathematical Notes on the Scale Free Networks

In this section, we briefly review some important results for the scale-free complex network; for more mathematical details see references [1, 2]. The starting point is definition of the degree distribution $p(k) = Ck^{-\gamma}$. The constant C can be calculated from normalization condition,

$$p(k) = \frac{(\gamma - 1)}{k_{min}} \left(\frac{k}{k_{min}} \right)^{-\gamma}. \tag{1}$$

In construction of a scale-free network with N nodes, the degrees are drawn from such a distribution. The largest degree, k_{max} statistically depends on the network size. This dependence can be calculated from the fact that the probability for having a node with degree exceeds the k_{max} is $1/N$.

$$\int_{k_{max}}^{\infty} p(k)dk = 1/N \tag{2}$$

Using 1 we obtain, $k_{max} = k_{min} N^{\frac{1}{\gamma-1}}$. It is worth noting again that this relation is statistically true and shows how k_{max} scales with N.

The average of the power of degree for this network is calculated from the following equation,

$$< k^m >= \int_{k_{min}}^{k_{max}} k^m p(k)dk = \frac{\gamma - 1}{\gamma - (m+1)} k_{min}^m \left[1 - \left(\frac{k}{k_{min}} \right)^{-\gamma+m+1} \right] \tag{3}$$

In region $\gamma > 2$, the average degree can be approximated by:

$$< k >= \frac{\gamma - 1}{\gamma - 2} k_{min} \tag{4}$$

While the average of the square power of degree, $< k^2 >$ is finite only for $\gamma \geq 3$ and diverges in $2 < \gamma < 3$ by increasing the network size,

$$\frac{< k^2 >}{< k >^2} = \frac{(\gamma - 2)^2}{(\gamma - 1)(\gamma - 3)} \left[1 - \left(\frac{\gamma - 1}{\gamma - 2} \frac{k_{max}}{< k >} \right)^{3-\gamma} \right] \tag{5}$$

In the above equation, we use 4 to replace k_{min} because in practice the average degree is used as an initial parameter for the construction of a network. The second term in bracket is a large value for $\gamma < 3$ while it can be ignored when γ is greater than three.

References

1. Barabási AL, et al. Network science. Cambridge university press; 2016.

2. Newman ME. Power laws, Pareto distributions and Zipf's law. Contemporary physics. 2005;46(5):323–351.
